# Caloric Restriction Shortens Lifespan through an Increase in Lipid Peroxidation, Inflammation and Apoptosis in the G93A Mouse, an Animal Model of ALS

**DOI:** 10.1371/journal.pone.0009386

**Published:** 2010-02-24

**Authors:** Barkha P. Patel, Adeel Safdar, Sandeep Raha, Mark A. Tarnopolsky, Mazen J. Hamadeh

**Affiliations:** 1 School of Kinesiology and Health Science, York University, Toronto, Ontario, Canada; 2 Muscle Health Research Centre, York University, Toronto, Ontario, Canada; 3 Department of Pediatrics, McMaster University, Hamilton, Ontario, Canada; 4 Department of Medicine, McMaster University, Hamilton, Ontario, Canada; 5 Department of Kinesiology, McMaster University, Hamilton, Ontario, Canada; Hospital Vall d'Hebron, Spain

## Abstract

Caloric restriction (CR) extends lifespan through a reduction in oxidative stress, delays the onset of morbidity and prolongs lifespan. We previously reported that long-term CR hastened clinical onset, disease progression and shortened lifespan, while transiently improving motor performance in G93A mice, a model of amyotrophic lateral sclerosis (ALS) that shows increased free radical production. To investigate the long-term CR-induced pathology in G93A mice, we assessed the mitochondrial bioenergetic efficiency and oxidative capacity (CS – citrate synthase content and activity, cytochrome *c* oxidase - COX activity and protein content of COX subunit- I and IV and UCP3- uncoupling protein 3), oxidative damage (MDA – malondialdehyde and PC – protein carbonyls), antioxidant enzyme capacity (Mn-SOD, Cu/Zn-SOD and catalase), inflammation (TNF-α), stress response (Hsp70) and markers of apoptosis (Bax, Bcl-2, caspase 9, cleaved caspase 9) in their skeletal muscle. At age 40 days, G93A mice were divided into two groups: Ad libitum (AL; n = 14; 7 females) or CR (n = 13; 6 females), with a diet equal to 60% of AL. COX/CS enzyme activity was lower in CR vs. AL male *quadriceps* (35%), despite a 2.3-fold higher COX-IV/CS protein content. UCP3 was higher in CR vs. AL females only. MnSOD and Cu/Zn-SOD were higher in CR vs. AL mice and CR vs. AL females. MDA was higher (83%) in CR vs. AL red *gastrocnemius*. Conversely, PC was lower in CR vs. AL red (62%) and white (30%) *gastrocnemius*. TNF-α was higher (52%) in CR vs. AL mice and Hsp70 was lower (62%) in CR vs. AL *quadriceps*. Bax was higher in CR vs. AL mice (41%) and CR vs. AL females (52%). Catalase, Bcl-2 and caspases did not differ. We conclude that CR increases lipid peroxidation, inflammation and apoptosis, while decreasing mitochondrial bioenergetic efficiency, protein oxidation and stress response in G93A mice.

## Introduction

Amyotrophic lateral sclerosis (ALS) is a neuromuscular disease characterized by the degeneration of motor neurons in the brain and spinal cord and is associated with an increase in oxidative stress [1], [2]. One mechanism which has been shown to decrease oxidative stress in animals and extend lifespan is caloric restriction (CR) [3]–[7]. Transgenic mice that overexpress the mutant human SOD1 gene (G93A mice) are an animal model of ALS and demonstrate elevations in free radical production [8]. Unexpectedly, when applied to this model, long-term CR hastened clinical onset, disease progression and life span, while transiently improving motor performance [9], [10]. Whereas short-term CR hastened clinical onset and shortened lifespan in male, but not female, G93A mice, and hastened disease progression with no effect on paw grip endurance [11]. The objective of this study was to explore how CR in the G93A mouse induces changes at the molecular level in genes involved in oxidative stress management, mitochondrial energetics, inflammation, stress response and apoptosis.

Unraveling the mechanism(s) behind the accelerated clinical onset and disease progression which occurs as a result of CR is necessary to understanding why this intervention failed to extend lifespan. This study is significant in determining the impact of food intake on the progression of disease in this animal model of ALS. We hypothesized that CR would decrease mitochondrial oxidative capacity which will induce electron leakage from mitochondrial electron transport chain resulting in an increase in oxidative stress, and hence markers of oxidative stress in the skeletal muscle of G93A mice. Furthermore, we hypothesized that CR would: (a) increase antixodant enzymes (MnSOD and Cu/Zn-SOD) and UCP3 as a compensatory mechanism to counteract the increase in oxidative stress, (b) not affect catalase activity since this enzyme is resistant to changes in dietaty intake, (c) increase TNF-α content, (d) decrease Hsp70 content, (e) increase Bax and Bcl-2 content, and the ratio of Bax/Bcl-2, as well as caspase 9, cleaved caspase 9 and the ratio of cleaved capase 9/caspase 9, signifying heightened apoptosis in this animal model and diminished protection from cellular stresses, such as inflammation, as a result of the elevated oxidative damage within the G93A mouse model.

## Materials and Methods

### Animals

Male B6SJL-TgN(SOD1-G93A)1Gur hemizygous mice (No. 002726) were harem-bred with female B6SJL nonaffected control mice (Jackson Laboratory, Bar Harbor, ME). The presence of the human G93A transgene was confirmed using PCR amplification of DNA extracted from tail samples as outlined by Jackson Laboratories. All animals were housed three to five per cage in a 12-h light/dark cycle. The experimental protocol strictly followed guidelines put forth by Canadian Council of Animal Care and McMaster University Animal Research Ethics Board. All necessary steps were taken to ameliorate suffering to animals involved in the study.

### Study Design

Eighty G93A mice (49 females, 31 males) were fed ad libitum (AL) after weaning (21 d) until the study commenced at age 40 d. At age 35 d, the mice were housed in individual cages. At age 40 d, 27 G93A mice (13 females, 14 males) were allocated to a surgery group and subsequently divided into two groups: the first group (AL; 7 females, 7 males) was provided food AL with a standard rodent diet (Harlan Teklad 22/5 Rodent Diet (W), product #8640); the second group (CR; 6 females, 7 males) was provided with 60% of the average intake of the AL group. The rest of the mice were also divided into AL (15 females, 7 males) and CR (21 females, 10 males) groups and carried to endpoint. The CR group diet (NIH-31/NIA Fortified Diet) was fortified with vitamins to ensure intakes similar to those of the AL group. The CR mice were provided with food equivalent to 60% of the average intake of the AL group on a daily basis. When mice attained a clinical score of 2.5 or started losing weight, food and calorie-free gel (Harlan-Gel, Harlan Teklad, Madison, WI) were placed on the floor of the cage to fulfill the requirements of the ethics committee. The calorie-free gel contained synthetic polymers (WATER LOCK® superabsorbent polymer G-400, G-430, G-500, G-530; ∼95% by weight) and methanol (<4.5% by weight). All measurements were conducted by two researchers. The researchers were not blinded to the treatment protocol. The inter-researcher coefficients of variation are 3.2% for body condition, 2.2% for ability to move and 0.24% for clinical score.

### Food Intake

Food intake measurements were initiated at age 43 d for all mice and recorded until age 95 d. The CR group was provided 60% of the average intake of the AL group, sex specific, following all measurements and testing.

### Body Weight and Body Condition

Body weights of mice were logged starting at age 43 d twice per week until age 99 d. Body weight and body condition for mice in the CR group were recorded before daily food portions were provided. Starting at age 43 d, body condition was assessed following a 5-point scale: 5 =  obese mice, 4 =  overconditioned mice (spine is a continuous column and the vertebrae are palpable only with firm pressure), 3 =  well-conditioned mice (the vertebrae and dorsal pelvis are not prominent and are palpable with slight pressure), 2 =  underconditioned mice (the segmentation of the vertebral column is evident and the dorsal pelvic bones are easily palpable), and 1 =  emaciated mice (the skeletal structure is extremely prominent and the vertebrae are distinctly segmented).

### Ability to Move

Starting at age 43 d, ability to move was assessed following a 5-point scale: 4 =  normal mobility, 3 =  move with limited use of the hindlegs, 2 =  move with the use of the forelegs, 1 =  move only for a short period with the use of the forelegs, and 0 =  unable to move. For the CR group, ability to move was recorded before mice were provided with their daily food portions.

### Paw Grip Endurance

Paw grip endurance (PaGE) was measured twice per week starting at age 70 d using the modified hanging wire test [12], [13]. Each mouse was placed on the wire-lid of a housing cage, placed at a height of 40 cm, and allowed to tightly hold on as induced by a gentle shake of the lid. Next, the lid was inverted and the time was recorded until the mouse fell off the lid, for a maximum of 90 seconds. This test was completed in triplicate, with the best result recorded. For the CR group, PaGE was recorded before mice were provided with food.

### Voluntary Activity

This study was part of a larger study investigating the effect of CR on oxidative stress. At age 100 d, muscle and tissue samples were harvested and stored at −80°C. This allowed us to measure voluntary activity in a representative subset of mice. Hence, in a subset of 64 mice (16 each for AL females, AL males, CR females and CR males), voluntary activity was recorded at age 72–89 d using mice activity wheels with living chambers (Single Activity Wheel Chamber Systerm Model 80820, Lafayette Instrument, Lafayette, IN). The mice were individually placed in the chambers and provided with food and water until they were returned to their cages. The activity wheel counters were interfaced with a computer which recorded cumulative activity in total revolutions over a 24-h period. Each 2.5 revolutions are equivalent to 1 m.

### Clinical Score

Using an 8-point scale, the clinical score for each mouse was assessed starting at age 81 d until age 95 d. The clinical score was dependent on signs exhibited to identify the severity of the disease: 0 =  no evidence of disease, 1 =  shaking of the hind legs or splaying of the hind legs when suspended by the tail (an indication of weakness in the hind legs), 1.5 =  weakness in one hindlimb (compensation for footdrop), 2 =  change in gait (used as clinical onset when attained on two consecutive days), 2.5 =  extreme weakness in one hindlimb (inability to dorsiflex), 3 =  extreme weakness in both hindlimbs, 3.5 =  functional paralysis in one hindlimb, 4 =  functional paralysis in both hind legs but can right themselves in less than 20 seconds after being placed on their side, and 5 =  cannot right themselves within 20 seconds after being placed on their sides (clinical endpoint). For the CR group, clinical score was recorded before the mice were provided with their daily food portions.

### Animal Sacrifice and Tissue Collection

Twenty seven G93A mice were sacrificed at ∼99 days of age, while the rest of the mice (n = 53) were led to endpoint. Mice were anesthetized with gaseous isoflurane and maintained under general anesthesia as the tissue was collected. Skeletal muscle (red and white *gastrocnemius*, and *quadriceps*) was removed and placed into individual sterile polyethylene tubes for immediate freezing in liquid nitrogen. All samples were stored at −86°C until analysis.

### Lipid Peroxidation

Red and white *gastrocnemius* was weighed, then minced and homogenized using a glass-Teflon Porter–Elvenhejm homogenizer (4% wt/vol) in buffer containing 50 mM dibasic potassium phosphate, 5 mM EDTA, 0.5 mM DTT, 1.15% KCl and 1 mM butylated hydroxytoluene at pH 7.4. The homogenates were centrifuged at 600 *g* for 10 min at 4°C. The resulting supernatant was decanted and protein concentrations of the spun muscle homogenates were determined using the Lowry Protein Assay. The homogenates were then assayed for malondialdehyde (MDA) according to Esterbauer and Cheeseman [14]. The absorbance of the supernatant was measured at 586 nm on an ultraviolet spectrophotometer (Cecil 9200 Super Aquarius, Cambridge, UK). A stock solution containing 10 mM 1,1,3,3,-tetramethoxypropane was used to obtain a standard curve of the following concentrations 0, 0.5, 1.0, 1.5, 2.5 and 5.0 µM, and the MDA concentrations in our samples were determined using the standard curve. All samples were analyzed in duplicate. MDA concentrations were expressed as nmol/g wet weight tissue.

### Protein Oxidation

Carbonyl groups were measured in red and white *gastrocnemius* using the Zentech Protein Carbonyl (PC) enzyme immunoassay kit (Zenith Technology, Dunedin, New Zealand). Samples containing protein were reacted with DNP, and nonspecifically adsorbed to an ELISA plate. Unconjugated DNP and nonprotein constituents were washed and the adsorbed protein was probed with a commercial biotinylated anti-DNP antibody followed by streptavidin-linked horseradish peroxidase. Absorbances were related to a standard curve (0, 0.1, 0.23, 0.46 and 1.07 nmol/mg) prepared of BSA containing increasing proportions of hypochlorous acid (HOCl)-oxidized protein that was calibrated colorimetrically. Absorbance was read at 450 nm on a fluorescence plate reader. Protein carbonyl concentrations were expressed as nmol/mg.

### Catalase Activity

Muscle catalase activity was determined by measuring the kinetic decomposition of H_2_O_2_, according to Aebi et al (1984) [15]. White *gastrocnemius* (50 µl) was added to an optical glass cuvette containing 930 µl of phosphate buffer (50 mM phosphate with 5 mM EDTA, and 0.1% Triton X-100 at pH 7.4). Then, 20 µl of 1 M H_2_O_2_ were added to the cuvette and mixed with a Pasteur pipette to initiate the reaction. Absorbance was measured at 240 nm for 2 min at 25°C. Catalase activity was calculated using the molar extinction coefficient for H_2_O_2_ of 0.0394 µmol/ml, and expressed in µmol H_2_O_2_/min/wet weight tissue.

### Citrate Synthase and Cytochrome *c* Oxidase Activity

Muscle lysate citrate synthase (EC 2.3.3.1) activity was determined by measuring the formation of thionitrobenzoate anion, as previously described [16]. Absorbance was recorded at 412 nm every 30 s for 3 min at 37°C. CS activity is expressed in nmol/min/mg of protein. Mitochondrial electron transport chain complex *c* oxidase (EC 1.9.3.1) activity was determined by measuring the rate of oxidation of reduced cytochrome *c* oxidase, as previously described [16]. Absorbance was recorded at 550 nm every 30 s for 3 min at 37°C. All samples were analyzed in duplicate on a spectrophotometer (Cary Bio-300, Varion, Inc., Palo Alto, CA).

### Western Blotting

Equal amounts of protein were size-separated by an 8–12.5% sodium dodecyl sulfate-polyacrylamide gel electrophoresis (SDS-PAGE) and were transferred to nitrocellulose membranes (#165-3322, Bio-Rad Mini-PROTEAN 3 electrophoresis system, Mississauga, ON, Canada) at 100 V. The membranes were blocked with 5% fat free milk or 3% BSA diluted in Tris-buffered saline with tween (0.5%) for 1 h at room temperature and incubated with primary antibodies against cytochrome *c* oxidase subunit I (COX-I; dilution 1∶1000; MS404, Mitosciences, Eugene, OR), cytochrome *c* oxidase subunit IV (COX-IV, 1∶3000; MS407, Mitosciences), citrate synthase (CS, 1∶4000; a generous gift from Dr. Brian Robinson, The Hospital for Sick Children, Toronto, ON), manganese superoxide dismutate (MnSOD, 1∶8000; ab13534, Abcam Inc., Cambridge, MA), copper/zinc superoxide dismuate (Cu/Zn-SOD, 1∶8000; ab16831, Abcam Inc.), uncoupling protein 3 (UCP3, 1∶1000; ab3477, Abcam Inc.), tumor necrosis factor – alpha (TNF-α, 1∶3500; ab9739, Abcam Inc.), heat shock protein 70 (Hsp70, 1∶500; MAB3846, Millipore), Bcl-2-associated X protein (Bax, 1∶3500; AB2915, Millipore), B-cell lymphoma 2 (Bcl-2, 1∶500; ab32124, Abcam Inc.) and caspase 9 & cleaved caspase 9 (1∶200; 9509, Cell Signalling Technology, Danvers, MA) overnight at 4°C or for 2 h at room temperature. Equal loading was ensured by ponceau staining as well as probing for GAPDH (1∶500 000; MAB374, Millipore). The antigen-antibody complexes were detected by incubating the membrane with anti-mouse (1∶10 000; AP160P, Millipore; COX-I, COX-IV, Hsp70, GAPDH) or anti-rabbit [(dilution 1∶5000; NA934V, Amersham, GE Healthcare, Baie d'Urfé, Quebec; CS, Cu/Zn SOD, MnSOD, UCP3), (1∶5000; AP307P, Millipore; TNF-α, Bcl-2, Bax) and (1∶2000; 7074, Cell Signalling; caspase 9, cleaved caspase 9)] HRP conjugated secondary antibodies at room temperature for 1 h. Immunoreactive proteins were visualized with enhanced chemiluminescence (RPN2132, Amersham, GE Healthcare; COX-I, COX-IV, CS, Cu/Zn SOD, MnSOD, and UCP3; or WBKLSO500, Millipore; TNF-α, Bcl-2, Bax, Hsp70, caspase 9, and cleaved caspase 9), and exposed to Kodak autoradiographic film (COX-I, COX-IV, CS, Cu/Zn SOD, MnSOD, and UCP3) or Progene autoradiographic film (TNF-α, Bcl-2, Bax, Hsp70, caspase 9, cleaved caspase 9). Films were scanned and the protein intensity was normalized to GAPDH using Image J (v 1.37, NIH, USA) from the NIH.

### Statistical Analysis

A three-way repeated measures ANOVA was used to determine significant differences in food intake, body weight, body condition, ability to move and PaGE, the between-subject factors being sex and diet (CR vs. AL), and the within-subject factor being time. This analysis was conducted on the 53 mice that were carried to endpoint. We conducted statistical analysis (3-way repeated measures ANOVA: 3 factors being sex, diet and time) and verified that the mice in the surgery group had outcome variables similar to the mice led to endpoint. No significant group effect was found for any of the following variables: body weight, body condition, ability to move, clinical onset and PaGE.

A two-way ANOVA was used to determine significant differences in MDA, PC, CS, COX and catalase enzyme activity, and protein content of MnSOD, Cu/Zn-SOD, UCP3, Hsp70, TNF-α, Bax, Bcl-2, caspase 9, cleaved caspase 9, the factors being sex and diet (CR vs. AL). When significance occurred (P≤0.05), a Tukey's HSD post hoc test was used to determine the source of the difference. One-tailed tests were used to determine differences between the diets within each sex for MDA and PC because we hypothesized *a priori* that oxidative stress would be higher in CR vs. AL mice. We hypothesized *a priori* that MnSOD, Cu/Zn-SOD and UCP3 would also increase as a compensatory response to the heightened oxidative stress, thus a one-tailed test was employed for these outcome measures. A one-tailed test was also used for Hsp70, TNF-α, Bax, Bcl-2, the ratio of Bax/Bcl-2, caspase 9, cleaved caspase 9 and the ratio of cleaved caspase 9/caspase 9 as we hypothesized *a priori* that inflammation and apoptosis would be elevated in CR vs. AL mice due to the inability of CR mice to respond to stress. All statistical analyses were completed using STATISTICA for Windows (version 6.0, StatSoft, Tulsa, OK). Data are presented as means ± standard error of mean (SEM). Significance was considered at P≤0.05.

## Results

### Food Intake

**Figure 1:**
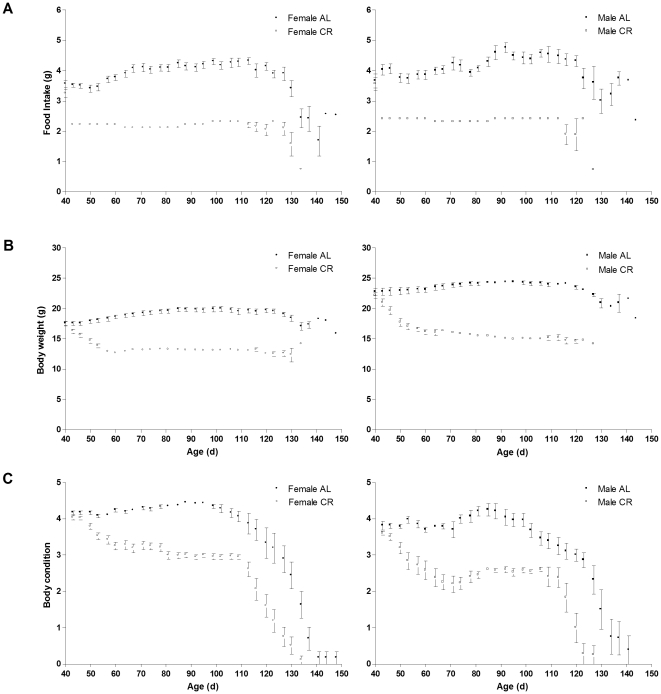
Anthropometric measures in CR vs. AL G93A mice. (A) Food intake (g), (B) body weight (g) and (C) body condition of 23 ad libitum (AL: •, 16 females; ▪, 7 males) and 31 calorie restricted (CR, 60% of ad libitum: ○, 21 females; □, 10 males) G93A mice. Data are means ± SEM.

For the CR mice, food intake before clinical onset was 2.2±0.0 g/d for females and 2.4±0.1 g/d for males, corresponding to 59% and 61% of the food intake for AL females and males, respectively (3.7±0.0 g/d for AL females and 3.9±0.1 g/d for AL males; Fig. 1).

### Body Weight

Body weight was lower in females vs. males (P<0.0001) and in CR vs. AL mice (P<0.0001). CR reduced the weight of mice by 23±1% for females and 25±2% for males within 10 days, after which body weight remained stable until close to endpoint (Fig. 1). From age 50 d to 70 d, the CR mice weighed 28% less than the AL mice (14±0 g vs. 20±1 g, respectively; P<0.0001), similar to values previously reported for CR mice [9], [17]–[20].

### Body Condition

Body condition was 35% lower in CR vs. AL mice (P<0.0001) and 15% lower in males vs. females (P<0.0001) (Fig. 1). For the AL mice, body condition was significantly lower after age 127 d as compared with baseline values (P≤0.008). For the CR mice, body condition significantly decreased within the 10 days immediately following CR, after which values were similar until age 106 d. Body condition after age 120 d was significantly lower as compared with values at age 53–106 d (P≤0.026).

### Ability to Move

**Figure 2:**
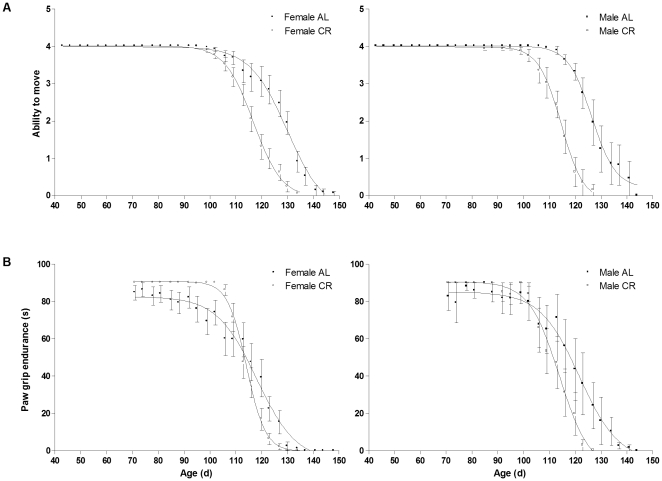
Functional measures in CR vs. AL G93A mice. (A) Ability to move and (B) paw grip endurance (s) of 23 ad libitum (AL: •, 16 females; ▪, 7 males) and 31 calorie restricted (CR, 60% of ad libitum: ○, 21 females; □, 10 males) G93A mice. Data are means ± SEM.

Ability to move was 14% lower in CR vs. AL mice (P<0.0001), with no sex differences (Fig. 2). For the AL mice, ability to move after age 130 d was significantly lower than baseline values (P≤0.016). For the CR mice, ability to move after age 109 d was significantly lower than baseline values (P≤0.004).

### Paw Grip Endurance

There were no sex and diet differences in PaGE (Fig. 2). At age 120 d and 123 d, PaGE was significantly lower in the CR vs. AL mice (diet x time interaction; P<0.032). For the AL mice, PaGE after age 120 d was significantly lower than baseline values (P≤0.031). For the CR mice, PaGE after age 113 d was significantly lower than baseline values (P<0.0001).

### Voluntary Activity

When voluntary activity was recorded, the age of mice was 82±1 d for AL females, 81±1 d for AL males, 81±1 d for CR females and 81±1 d for CR males (not significant). Voluntary activity per 24 h was 18,528±1850 revolutions (7411±740 m) for AL females, 8642±1459 revolutions (3457±584 m) for AL males, 25,299±3121 revolutions (10,120±1248 m) for CR females and 21,504±2649 revolutions (8602±1060 m) for CR males. Females were 45% more active than males (P = 0.007), whereas CR mice were 72% more active than AL mice (P = 0.0002).

### Clinical Onset

**Figure 3:**
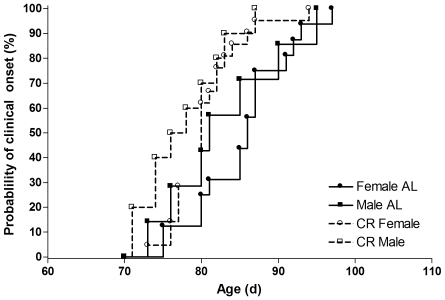
Probablility of clinical onset in CR vs. AL G93A mice. Probability of clinical onset in 23 ad libitum (AL: •, 16 females; ▪, 7 males) and 31 calorie restricted (CR, 60% of ad libitum: ○, 21 females; □, 10 males) G93A mice. The rate of attaining clinical onset (i.e., the hazard ratio) in the CR mice was 2-fold higher (95% CI: 1.4, 4.7) than the AL mice. CR females had a 2-fold (95% CI: 1.2, 5.3) higher rate of attaining clinical onset as compared with AL females (P = 0.016). There was no sex difference in the rate of attaining clinical onset.

Clinical score was significantly higher in CR vs. AL mice (P<0.0001). The age of mice at clinical onset was 85±1 d for AL mice and 80±1 d for CR mice (P = 0.005), 5 days younger for the CR mice, a 6% difference. Males showed a trend for reaching clinical onset 3 days younger than females (P = 0.096; females, 83±1 d; males, 80±2 d). The clinical onset curves were significantly different between the groups (Logrank test, P = 0.003), with the rate of attaining clinical onset (i.e., the hazard ratio) in the CR mice being 2-fold higher (95% CI: 1.4, 4.7) than the AL mice (Fig. 3). CR females had a 2-fold (95% CI: 1.2, 5.3) higher rate of attaining clinical onset as compared with AL females (P = 0.016). There was no sex difference in the rate of attaining clinical onset.

### Disease Progression

There was no sex difference in disease progression for either endpoint (clinical score of 4 or 5). Using clinical score of 4 as endpoint, progression of the disease from clinical onset to endpoint was 8 d faster for the CR (37±2 d) vs. AL (45±3 d) mice (P = 0.011), an 18% difference. Using clinical score of 5 as endpoint, progression of the disease from clinical onset to endpoint was 6 d faster for the CR (42±1 d) vs. AL (48±3 d) mice (P = 0.014), a 13% difference.

**Figure 4:**
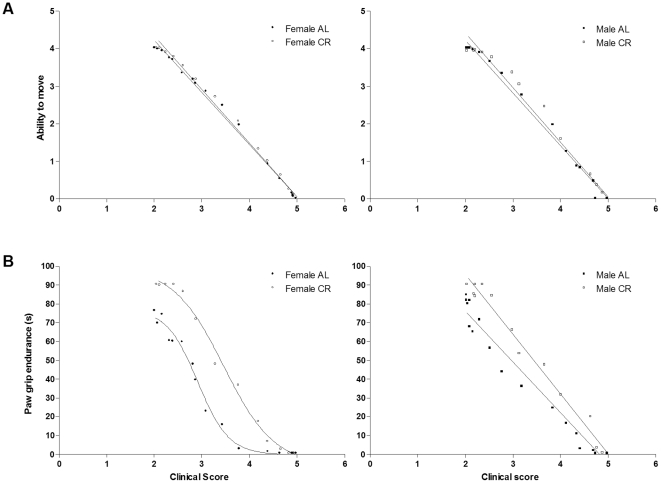
Correlation between clinical score and ability to move and between clinical score and paw grip endurance in CR vs. AL G93A mice. (A) Relation between clinical score (from clinical onset to euthanasia) and ability to move and (B) between clinical score and paw grip endurance (s) in 23 ad libitum (AL: •, 16 females; ▪, 7 males) and 31 calorie restricted (CR, 60% of ad libitum: ○, 21 females; □, 10 males) G93A mice. For ability to move, slopes and intercepts were not different with the following pooled equation: ability to move  =  (−1.4) x clinical score + (7.1). For paw grip endurance, data for the females followed a sigmoidal relationship (AL females, r^2^ = 0.990; CR females, r^2^ = 0.995; curves were significantly different, P<0.0001) whereas data for the males followed a linear relationship (slopes were significantly different, P = 0.0011). For AL males (r = −0.984, P<0.0001), paw grip endurance  =  (−27.8±1.1) x clinical score + (133.5±3.8); for CR males (r = −0.995, P<0.0001), paw grip endurance  =  (−31.8±0.7) x clinical score + (159.4±2.9), with the data presented as means ± SD. Data are means of each group on the same day.

From clinical onset (clinical score of 2) to euthanasia, ability to move strongly and negatively correlated with clinical score (AL females, r = −0.997; AL males, r = −0.996; CR females, r = −0.997; CR males, r = −0.994) (Fig. 4). There was no difference between the curves.

There was a strong relation between clinical score and PaGE (Fig. 4). For the females, the relationship was sigmoidal (AL females, r^2^ = 0.990; CR females, r^2^ = 0.995; curves were significantly different, P<0.0001). For the males, the relationship was linear (AL males, r = −0.984; CR males, r = −0.995; slopes were significantly different, P = 0.0011).

### Survival

**Figure 5:**
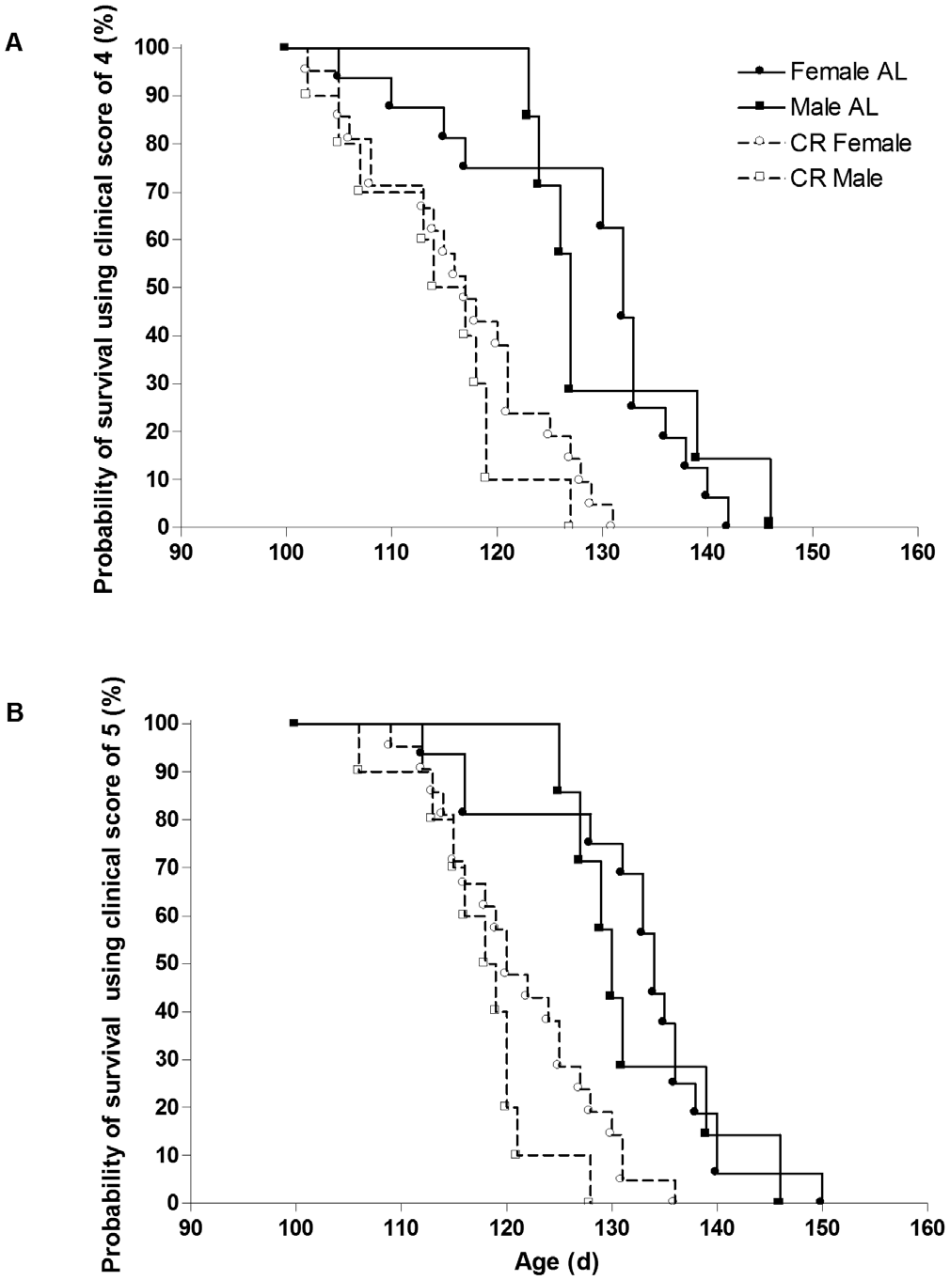
Probability of survival in CR vs. AL G93A mice. Probability of survival using as endpoint a clinical score of (A) 4 and (B) 5 in 23 ad libitum (AL: •, 16 females; ▪, 7 males) and 31 calorie restricted (CR, 60% of ad libitum: ○, 21 females; □, 10 males) G93A mice. The rate of reaching endpoint in the CR mice (i.e., the hazard ratio) was 3.1-fold higher (95% CI: 2.9, 10.7) than the AL mice. The rate of reaching endpoint was 3.1-fold (95% CI: 2.6, 13.5) higher in the CR vs. AL females (P<0.0001) and 3.4-fold (95% CI: 1.9, 20.7) higher in the CR vs. AL males (P = 0.003).

There was no sex difference in survival for either endpoint (clinical score of 4 or 5). The age of mice at clinical score of 4 was 129±2 d for the AL mice and 116±1 d for the CR mice (P<0.0001), 13 d younger for the CR mice. This is equivalent to a 10% decrease in life span. Survival curves were significantly different between the groups (P<0.0001; Fig. 5), with the rate of reaching endpoint in the CR mice (i.e., the hazard ratio) being 3.1-fold higher (95% CI: 2.9, 10.7) than the AL mice. The rate of reaching endpoint was 3.1-fold (95% CI: 2.6, 13.5) higher in the CR vs. AL females (P<0.0001) and 3.4-fold (95% CI: 1.9, 20.7) higher in the CR vs. AL males (P = 0.003).

The age of mice at clinical score of 5 was 132±2 d for the AL mice and 120±1 d for the CR mice (P<0.0001), 12 days younger for the CR mice. This is equivalent to a 9% decrease in life span. Survival curves were significantly different between the groups (P<0.0001; Fig. 5), with the rate of reaching endpoint in the CR mice (i.e., the hazard ratio) being 3.1-fold higher (95% CI: 2.6, 9.8) than the AL mice. The rate of reaching endpoint was 2.9-fold (95% CI: 2.1, 10.2) higher in the CR vs. AL females (P = 0.0001) and 4-fold (95% CI: 2.8, 34.2) higher in the CR vs. AL males (P = 0.0004).

There were no significant differences in the anthropometric data between the group which was followed to endpoint vs. the group that was sacrificed at age 99 d.

### Mitochondrial Oxidative Capacity

**Figure 6:**
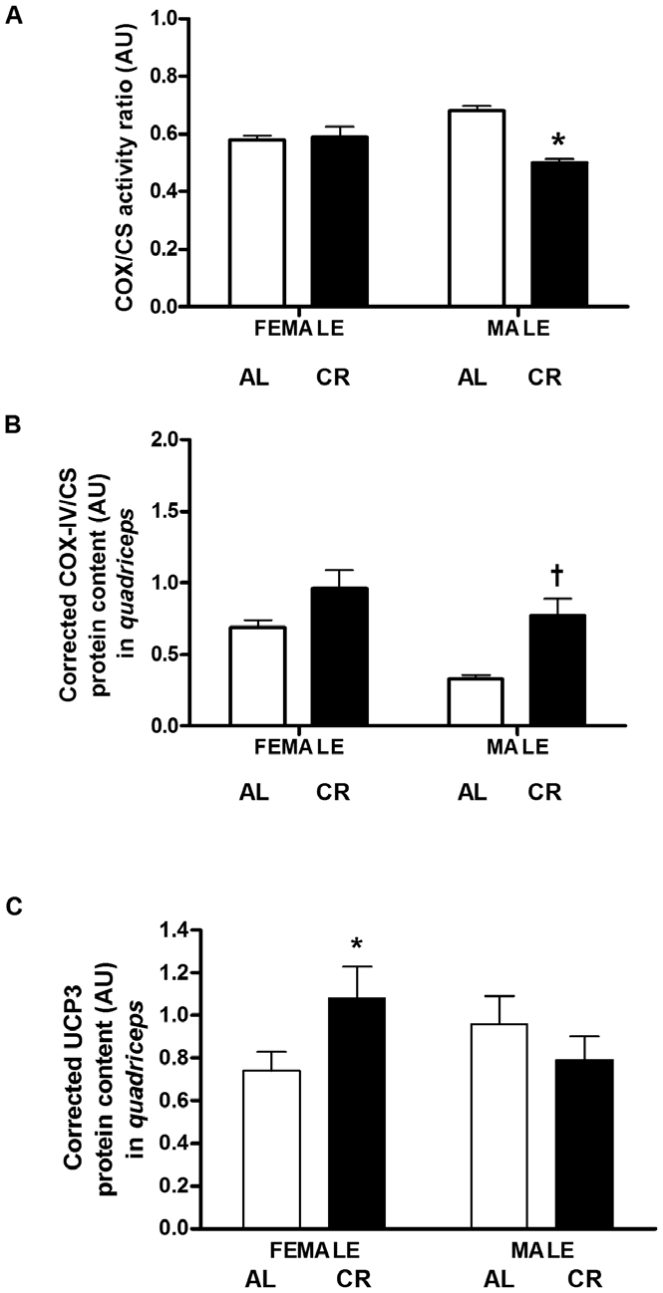
Mitochondrial energetics in CR vs. AL G93A mice. (A) COX to citrate synthase activity ratio was lower in the *quadriceps* of CR *vs.* AL G93A male mice (34%, P = 0.034). (B) Cytochrome *c* oxidase subunit-IV to citrate synthase protein content ratio was higher in CR *vs.* AL G93A male mice (2.3-fold, P = 0.059). (C) UCP3 was higher in CR vs. AL female *quadriceps* (1.5-fold, P = 0.042). Data are presented as means ± SEM. n = 27; AL, 7 males and 7 females; CR, 7 males and 6 females. Asterisks denote significant changes (P≤0.05 vs. AL); dagger denotes strong trend (0.05<P≤0.1 vs. AL male).

In *quadriceps*, there were no differences in CS protein content and enzyme activity, cytochrome *c* oxidase subunit- I and IV protein content and COX activity between CR and AL or female and male mice (data not shown). However, COX to citrate synthase activity ratio was lower in CR vs. AL males (34%, P = 0.034; Fig. 6, panel A). Interestingly, cytochrome *c* oxidase subunit-IV to citrate synthase protein content ratio was higher in CR vs. AL males (2.3-fold, P = 0.059; Fig. 6, panel B). The protein content of UCP3 was significantly higher in CR vs. AL females (1.5-fold, P = 0.042; Fig. 6, panel C). However, unlike the CR females, UCP3 protein content was not significantly higher in CR vs. AL males.

### Lipid Peroxidation

**Figure 7:**
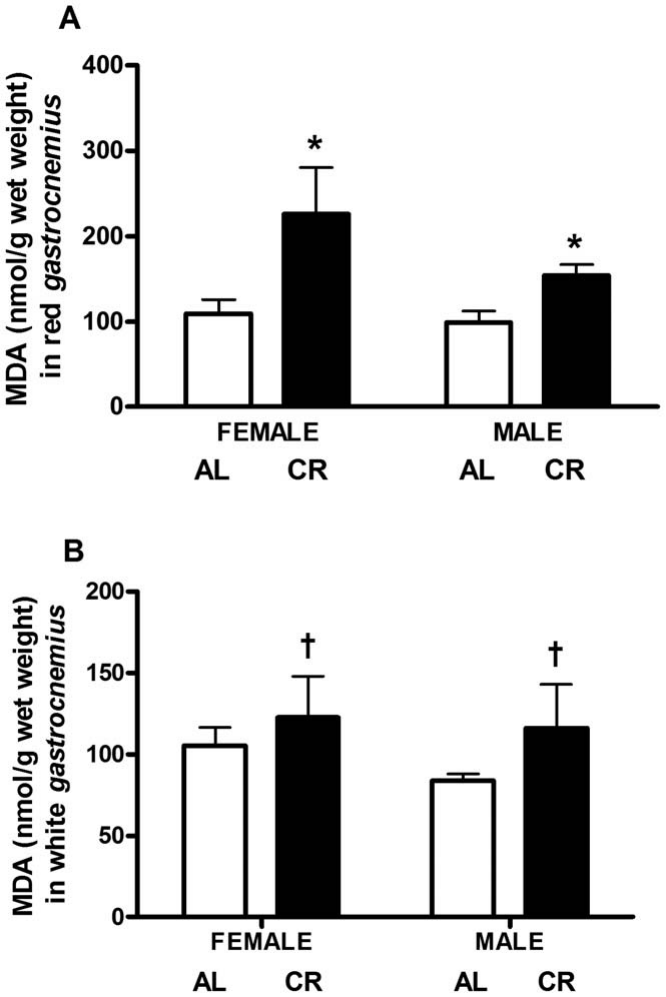
Lipid peroxidation in CR vs. AL G93A mice. (A) MDA was higher in CR vs. AL (83%, P<0.001; main effect of diet), and in female vs. male (32%, P = 0.045; main effect of sex) red *gastrocnemius*. Within each sex, MDA was higher in CR vs. AL females (2-fold, P = 0.003), and in CR vs. AL males (56%, P = 0.005). (B) MDA was higher in CR vs. AL white *gastrocnemius* (14%, P = 0.095; main effect of diet), with no sex differences. Data are presented as means ± SEM. Asterisks denote significant changes (P≤0.05 vs. AL); dagger denotes strong trend (0.05<P≤0.1 vs. AL).

Spectrophotometric analysis of lipid peroxidation in red and white *gastrocnemius* revealed that MDA, a proxy measure of lipid damage, was higher in both female and male mice under CR vs. AL. MDA was higher in red *gastrocnemius* (83%, P<0.001; Fig. 7, panel A) as a result of CR, with females having higher levels than males (32%, P = 0.045). Within each sex, MDA was higher in CR vs. AL females (2-fold, P = 0.003), and in CR vs. AL males (56%, P = 0.005). In the white *gastrocnemius*, MDA was higher (14%, P = 0.095; Fig. 7, panel B) due to CR, with no sex differences.

### Protein Oxidation

**Figure 8:**
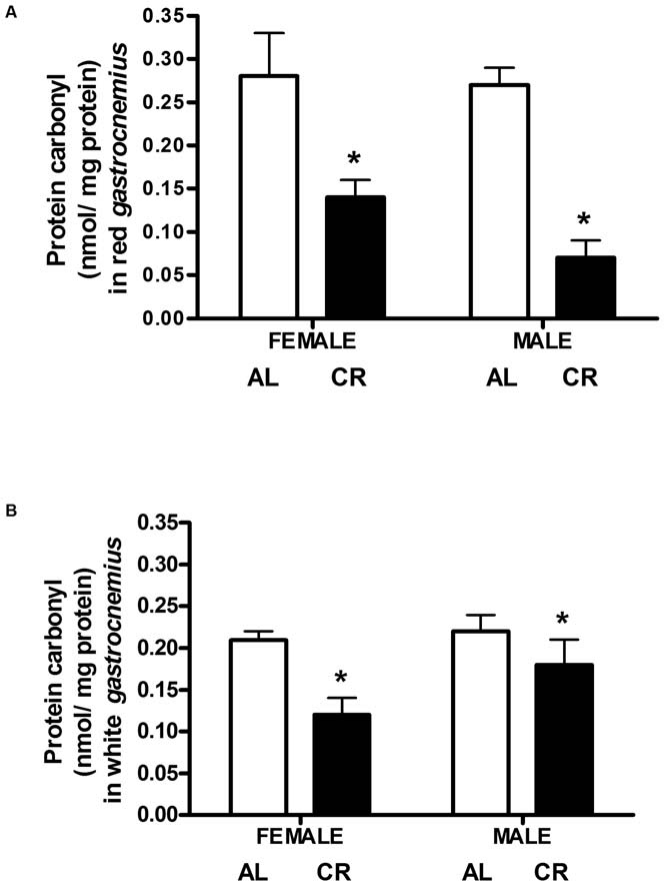
Potein oxidation in CR vs. AL G93A mice. (A) PC was lower in CR vs. AL red *gastrocnemius* (62%, P<0.001; main effect of diet). Within each sex, PC was lower in CR vs. AL females (50%, P = 0.012), and in CR vs. AL males (74%, P<0.001). (B) PC was lower in CR vs. AL white *gastrocnemius* (30%, P = 0.003; main effect of diet). PC was lower in CR vs. AL females (43%, P<0.001). Data are presented as means ± SEM. n = 27; AL, 7 males and 7 females; CR, 7 males and 6 females. Asterisks denote significant changes (P≤0.05 vs. AL).

PC, an indication of oxidative damage to proteins, did not increase as a result of CR. In the red *gastrocnemius*, CR significantly lowered PC (62%, P<0.001; Fig. 8, panel A), with no sex differences. Within each sex, PC was lower in CR vs. AL females (50%, P = 0.012), and in CR vs. AL males (74%, P<0.001). In the white *gastrocnemius*, CR lowered PC (30%, P = 0.003; Fig. 8, panel B), with no sex differences. PC was lower in CR vs. AL females (43%, P<0.001) only.

### Antioxidant Enzymes

**Figure 9:**
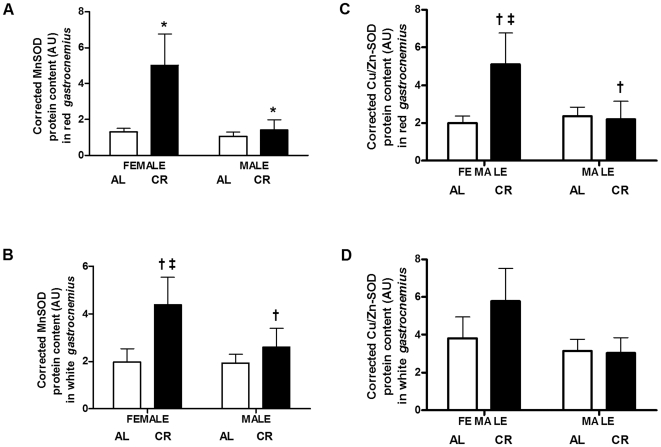
SOD content in CR vs. AL G93A mice. (A) MnSOD was higher in CR vs. AL (3-fold, P = 0.031; main effect of diet), and in female vs. male (2.5-fold, P = 0.026; main effect of sex) red *gastrocnemius*. MnSOD was significantly elevated in CR vs. AL females (4-fold, P = 0.015), but not in CR vs. AL males. (B) MnSOD was higher in CR vs. AL white *gastrocnemius* (78%, P = 0.062; main effect of diet). MnSOD was higher in CR vs. AL females (2.2-fold, P = 0.038). (C) Cu/Zn-SOD was higher in CR vs. AL red *gastrocnemius* (67%, P = 0.096; main effect of diet). Cu/Zn-SOD was significantly higher in CR vs. AL females (2.6-fold, P = 0.020). (D) There was no change in Cu/Zn-SOD protein content in white *gastrocnemius*. Data are presented as means ± SEM. n = 27; AL, 7 males and 7 females; CR, 7 males and 6 females. Asterisks denote significant changes (P≤0.05 vs. AL); dagger denotes strong trend (0.05<P≤0.1 vs. AL); double-dagger denotes significant changes (P≤0.05 vs. AL females).

The protein content of the antioxidant enzyme MnSOD displayed a significant upregulation in red *gastrocnemius*. MnSOD was significantly higher in CR vs. AL red *gastrocnemius* (3-fold, P = 0.031; Fig. 9, panel A), with a substantial upregulation in the protein content in females vs. males (2.5-fold, P = 0.026). An interaction between diet and sex revealed that CR females were significantly different from CR males (P = 0.038), AL females (P = 0.031) and AL males (P = 0.020). Within each sex, MnSOD was significantly elevated in CR vs. AL females (4-fold, P = 0.015), but not in CR vs. AL males. In the white *gastrocnemius*, MnSOD was higher in CR vs. AL mice (78%, P = 0.062; Fig. 9, panel B), with no sex differences. MnSOD was higher in CR vs. AL females (2.2-fold, P = 0.038) only. Despite the upregulation in the protein content of MnSOD, Cu/Zn-SOD, another potent antioxidant enzyme, was only up-regulated in red gastrocnemius. Cu/Zn-SOD was higher in CR vs. AL red gastrocnemius (67%, P = 0.096; Fig. 9, panel C), with no sex differences. Cu/Zn-SOD was significantly higher in CR vs. AL females (2.6-fold, P = 0.020). In the white gastrocnemius, there was no change in Cu/Zn-SOD protein content (Fig. 9, panel D).

**Figure 10:**
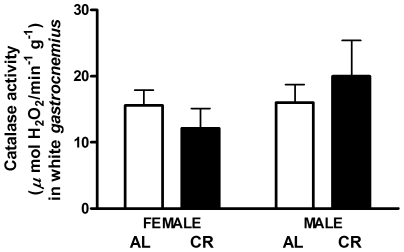
Catalase activity in CR vs. AL G93A mice. Catalase enzyme activity remained unchanged in the white *gastrocnemius* of G93A mice. Data are presented as means ± SEM. n = 27; AL, 7 males and 7 females; CR, 7 males and 6 females.

Catalase enzyme activity was not measured in red gastrocnemius due to insufficient amounts of tissue. In white gastrocnemius, catalase activity, measured from the kinetic decomposition of H_2_O_2_, was not different between female and male mice under CR or AL (Fig. 10).

### Protein Content of TNF-α – Marker of Inflammation

**Figure 11:**
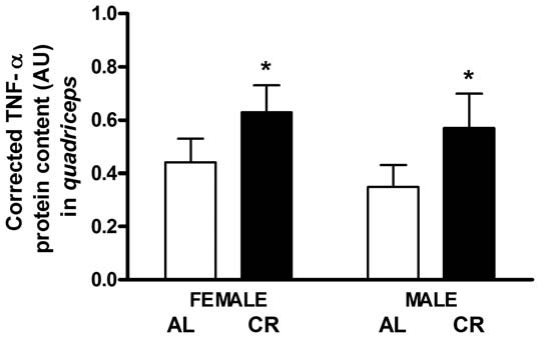
TNF-α content in CR vs. AL G93A mice. TNF-α was higher in the *quadriceps* of CR vs. AL mice (52%, P = 0.030; main effect of diet). Data are presented as means ± SEM. n = 27; AL, 7 males and 7 females; CR, 7 males and 6 females. Asterisks denote significant changes (P≤0.05 vs. AL).

TNF-α, a marker of inflammation, was significantly higher in the *quadriceps* of CR vs. AL mice (52%, P = 0.030; Fig. 11). There were no sex differences in the protein content of TNF-α.

### Protein Content of Genes Related to Cell Stress Response and Apoptosis

**Figure 12:**
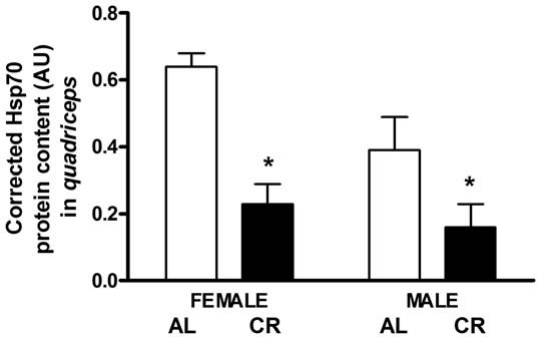
Hsp70 content in CR vs. AL G93A mice. Hsp70 was lower in the *quadriceps* of CR vs. AL mice (62%, P = 0.002; main effect of diet), and in males vs. females (37%, P = 0.030; main effect of sex). Within each sex, Hsp70 was significantly decreased in CR vs. AL females (64%, P<0.001) and in CR vs. AL males (59%, P = 0.039). Data are presented as means ± SEM. n = 27; AL, 7 males and 7 females; CR, 7 males and 6 females. Asterisks denote significant changes (P≤0.05 vs. AL).

The protein content of a molecular chaperone, Hsp70, generally up-regulated in response to cell stress, was reduced as a result of CR. In the *quadriceps*, Hsp70 was significantly lower in CR vs. AL (62%, P = 0.002; Fig. 12) and in male vs. female (37%, P = 0.030) mice. Within each sex, Hsp70 was significantly lower in CR vs. AL females (64%, P<0.001) and in CR vs. AL males (59%, P = 0.039).

**Figure 13:**
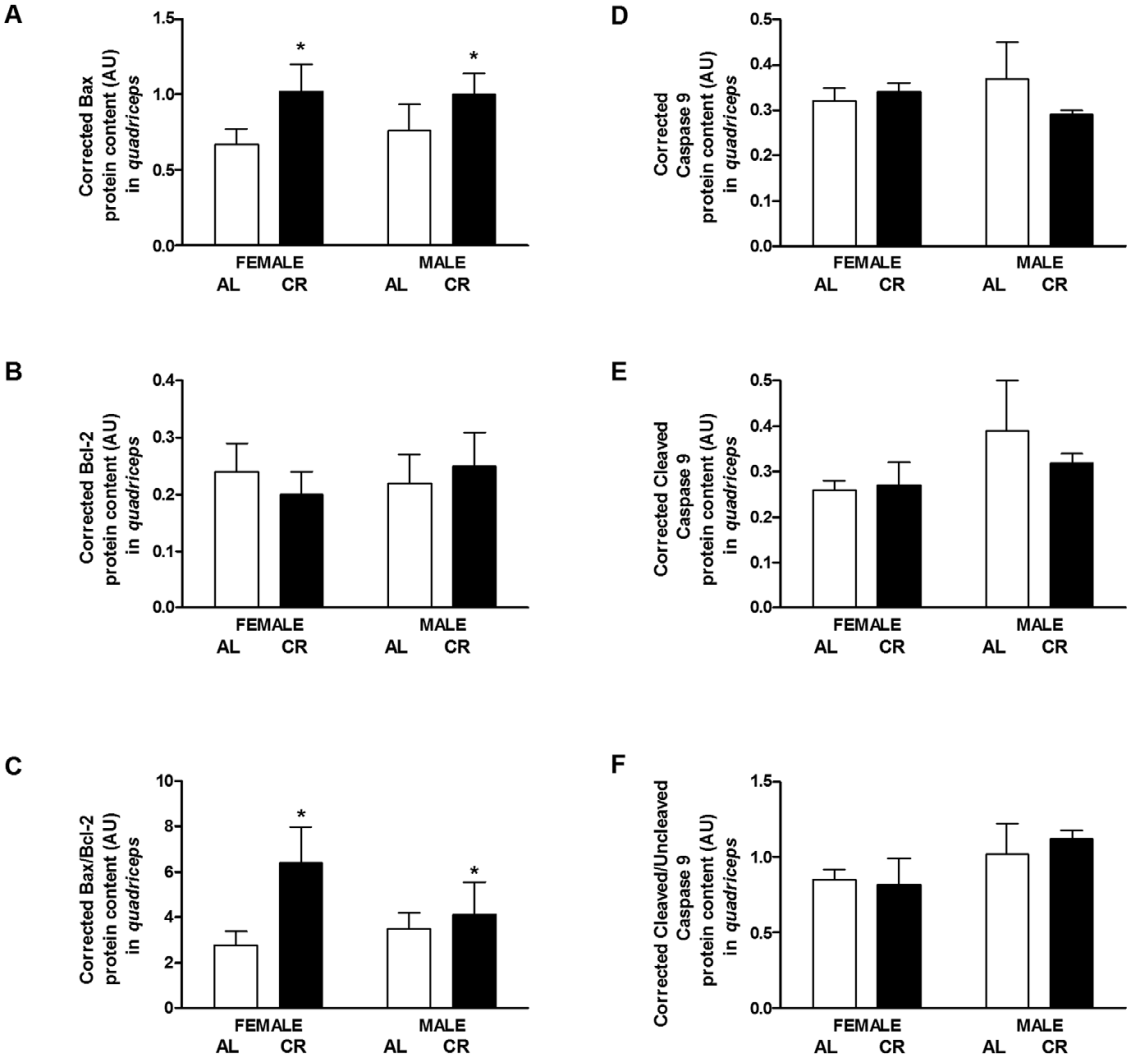
Apoptosis regulatory proteins content in the *quadriceps* of CR vs. AL G93A mice. (A) Bax was significantly higher in CR vs. AL mice (41%, P = 0.027; main effect of diet). Bax was increased in CR vs. AL females (52%, P = 0.048). (B) There was no significant difference in Bcl-2 protein content. (C) Bax/Bcl-2 was significantly higher in CR vs. AL mice (68%, P = 0.040; main effect of diet). Bax/Bcl-2 was increased in CR vs. AL females (2.3-fold, P = 0.029). No significant changes were observed in the protein content of: (D) caspase 9, (E) cleaved caspase 9, and (F) the ratio of cleaved caspase 9/caspase 9 for diet or sex. Data are presented as means ± SEM. n = 27; AL, 7 males and 7 females; CR, 7 males and 6 females. Asterisks denote significant changes (P≤0.05 vs. AL).

In relation to the heightened inflammation and decreased ability to respond to stress under CR, Bax, a pro-apoptotic protein, was elevated in CR mice. Bax was significantly higher in CR vs. AL mice (41%, P = 0.027; Fig. 13, panel A) and in CR vs. AL females (52%, P = 0.048). While there was no increase in the protein content of Bcl-2, an anti-apoptotic protein in the *quadriceps* (Fig. 13, panel B), the ratio of Bax/Bcl-2 protein content indicated heightened apoptosis under CR. Bax/Bcl-2 was significantly higher in CR vs. AL mice (68%, P = 0.040; Fig. 13, panel C), and in CR vs. AL females (2.3-fold, P = 0.029) only.

During apoptosis, caspase 9 is activated to form cleaved caspase 9. However, we did not observe any changes in the protein content of caspase 9 (Fig. 13, panel D), cleaved caspase 9 (Fig. 13, panel E) or the ratio of cleaved caspase 9/caspase 9 for diet or sex (Fig. 13, panel F)

## Discussion

This is the first study to illustrate that CR is detrimental. In the current study, CR equivalent to 60% of AL hastened clinical onset of disease, disease progression and endpoint in the G93A mouse, an animal model of ALS. We also ascertained that using a clinical score of 4 as a proxy measure of endpoint would result in similar conclusions as using a clinical score of 5. Both the female sex and CR induced an increase in 24-h voluntary activity.

We examined the effect of long-term CR on markers of mitochondrial bioenergetics, oxidative damage, antioxidant enzyme capacity, inflammation, stress response and apoptosis in the skeletal muscle of G93A mice, an animal model of ALS. In this study, we ascertained that CR reduced the ratio of COX to citrate synthase activity in male G93A mice despite an up-regulation of the ratio of COX subunit-IV to citrate synthase protein content. Contrarily, CR females upregulated UCP3, a marker of mitochondrial energetics, which is activated in response to enhanced oxidative stress [21]. CR increased lipid peroxidation, but decreased protein oxidation. The increase in oxidative stress was accompanied by a substantial upregulation in the protein content of antioxidant enzymes MnSOD and Cu/Zn-SOD in CR vs. AL females. This upregulation in antioxidant enzymes was not observed in CR males. Furthermore, CR increased markers of inflammation and apoptosis, while decreasing Hsp70, a stress response protein.

The life-extending properties of CR have been observed in insects, rodents and non-human primates, and are mainly attributed to a reduction in mitochondrial oxidant production and an increase in mitochondrial bioenergetic efficiency [3]–[7]. However, nutritional status in ALS patients is predictive of survival [22], with a low energy intake significantly correlating with death in this population [23], [24]. Research involving G93A mice, the animal model of ALS used in this study, has provided strong evidence for the negative implications of reduced energy intake in the pathogenesis of disease [25], specifically that long-term CR hastens the clinical onset of disease, disease progression and life span in G93A mice [9], [10]. However, the molecular basis of the negative effects of CR on G93A mouse lifespan and survival remained undetermined. In the present study, we investigated the effect of CR on markers of mitochondrial bioenergetics, oxidative stress, antioxidant enzyme capacity, inflammation, stress response and apoptosis.

CR induces mitochondrial biogenesis and bioenergetic efficiency in both animals and humans [26]–[28]. In the current study, we observed that CR significantly increased the ratio of COX-IV to citrate synthase protein content in male G93A mice. However, this increase did not translate into enhanced mitochondrial oxidative capacity since the ratio of COX to citrate synthase activity (marker of mitochondrial bioenergetic efficiency) is reduced in CR male G93A mice. We propose that CR increases the protein content of subunits of mitochondrial repiratory chain in male G93A mice to compensate for reduced mitochondrial bioenergetic efficiency. Interestingly, patients with primary mitochondrial myopathy as a result of point mutation in their mitochondrial genome show similar increases in mitochondrial content without an adaptive increase in mitochondrial function [29]–[31]. The discordant effects of CR observed on mitochondrial content vs. function suggest that the mitochondrial genome may have been compromised due to higher levels of oxidative stress in G93A mice as previously reported [31]–[34], and that an increase in mitochondrial biogenesis in response to CR [25]–[27] only results in accumulation of dysfunctional mitochondrial proteins.

Furthermore, it has been shown that 95 d old G93A mice have significantly elevated levels of MDA (marker of lipid peroxidation) and PC (markers of protein oxidation) in skeletal muscle, as well as a substantial increase in MnSOD, Cu/Zn-SOD and catalase enzyme activity compared with wild-type mice [32]. Thus, G93A mice have a basal increase in oxidative stress due to the overexpression of its mSOD1 protein [32]–[35]. In the present study, we determined that CR significantly increased MDA in both the red and white gastrocnemius. It is possible that decreases in mitochondrial oxidant production, as a result of long-term CR, failed to balance the increase in free radical production from the mutant enzyme found mainly in the cytosol, leading to a net increase in oxidative stress [9]. One short-term CR study showed a similar pattern, with significantly higher lipid peroxidation potential in the hepatic microsomes of slightly restricted (75% of AL) young (3.5 mo) mice compared with severely restricted (50% of AL) young mice, slightly and severely restricted old (27 mo) mice, and severely restricted senescent (45 mo) mice [36]. Lipid peroxidation potential, induced by the addition of Fe^2+^, was determined by the presence of lipid hydroperoxides (LOOH) in the microsomes [36]. After the addition of increasing concentrations of Fe^2+^, the severely restricted young mice and the slightly and severely restricted old mice exhibited the lowest lipid peroxidation potential, whereas the lipid peroxidation potential in the severely restricted senescent mice increased to intermediate levels (approximately 60% lower vs. the slightly restricted young) [36]. Surprisingly, the slightly restricted young mice exhibited the highest lipid peroxidation potential. These results indicate that severe short-term CR in the young and old mice lowered lipid peroxidation potential, but in the senescent mice this effect was attenuated as a result of age. Thus, the magnitude of energy restriction, as well as age, have an impact on the peroxidative potential of hepatic microsomes [36]. The authors suggest that severe CR has a greater effect in reducing lipid peroxidation earlier in life [36], hence the higher peroxidative potential in the young slightly restricted mice. In confirmation, a previous study found that dietary restriction of 55 kcal/wk significantly reduced lipid peroxidation in 12-month old mouse liver, but not in 24-month old restricted mice [37]. Therefore, the CR-induced reduction in lipid peroxidation can be attenuated by age.

Short-term CR (2 mo; ∼60% of AL) in male Fisher 344 rats showed decreased H_2_O_2_ production (14% reduction) and a diminution in mitochondrial antioxidant enzyme activities (SOD2, 39% reduction; GPx, 19% reduction), but increased PC in cardiac muscle as compared with AL rats [38]. Judge et al (2003) postulated that the increase in PC may be a result of the reduction in SOD2 and GPx activity and/or that protein oxidation is transiently elevated in cardiac muscle [38]. Furthermore, CR (2 mo; 70% of AL) increased MDA by 3-fold in the liver and by 50% in the heart of male Sprague-Dawley rats, an animal model with elevated basal levels of stress-induced corticosterone, vs. AL-fed rats [39]. Conversely, Gredilla et al (2001) established that 6 weeks of CR (60% of AL) significantly decreased mitochondrial and nuclear marker of DNA damage, 8-OHdG, and H_2_O_2_ production in the liver of male Wistar rats [40]. The elevation we observed in MDA in the skeletal muscle of G93A mice, which exhibit increased basal levels of oxidative stress [32], is in accordance with the heightened oxidative damage observed in the study by Gursoy et al (2000), which used another animal model with higher basal levels of stress [39]. An increase in oxidative stress under short-term CR depends on the tissue used, age of animal model, duration of restriction, magnitude of restriction and rodent species analyzed. In our G93A mice, 2 mo of CR (60% of AL) increased peroxidative damage to lipids, indicating heightened oxidative stress.

In contrast to the effect of CR on increasing MDA, protein oxidation following CR is decreased in G93A mice. We determined that CR significantly decreased PC compared with AL mice. This finding is in accordance with another study using similar energy restriction (60% of AL), whereby CR prevented the age-associated increase in PC in mouse hindlimb skeletal muscle mitochondria [41]. In our study, MDA and PC move in opposing directions as a consequence of CR, which is a finding that proposes some important questions, including whether an increase in PC is concordant with an increase in MDA. A study by Sato el al (1998) revealed that PC levels approximate the unsaturation of lipids in the muscle at opposite extremes, but not in the brain, heart or liver, and although the study did not measure lipid peroxidation, it however reported the peroxidizability and unsaturation indexes (PI and UI, respectively) of each lipid used [42]. Stroke-prone spontaneously hypertensive rats (SHRSP) were fed a diet containing either lard, safflower oil, perilla oil or a fish oil/soybean oil combination [42]. The PI and UI values increased from lard to fish/soybean oil when examining the fatty acid composition of each diet. However, these PI and UI values were altered in vivo depending on the specific tissue analyzed. PC levels in the brain were significantly higher for lard vs. fish/soybean oil only, results inconsistent with the lack of significant difference in the PI or UI in the brain in vivo. In the heart, the fish/soybean oil had a higher PI and UI vs. all other oils, yet PC levels were indistinguishable between the diets. In the liver, the fish/soybean oil group exhibited significantly higher PI and UI vs. all other groups. However, PC content in the liver was higher in the lard diet vs. all other diets, with no difference in PC content between the other oils. In skeletal muscle, fish/soybean oil had significantly higher UI and PI values vs. all other groups, whereas the UI for lard was significantly lower than all other groups. The skeletal muscle PC content in the lard group was significantly lower vs. the safflower, perilla and fish/soybean oil groups, but there was no significant difference in PC content between safflower, perilla and fish/soybean oil groups [42]. These finding indicate that PC and lipid peroxidation levels do not necessarily change in parallel, but are dependent on the tissue and degree of unsaturation. In addition, the variations in the PI and UI of the oils were not reflected proportionately in every tissue. In skeletal muscle, although PC increased in mice fed fish/soybean oil as compared with lard, this increase was similar to mice fed safflower and perilla oils [42]. Hence, even within skeletal muscle, the PI or UI does not dictate tissue PC levels.

To further support this, Sen et al (1997) reported that fish oil vs. soy oil supplementation increased lipid peroxidation in the liver, but not in the red gastrocnemius of male Wistar rats [43]. However, fish oil did not induce protein oxidative damage in the liver or red gastrocnemius. There was no correlation between markers of lipid peroxidation and markers of protein oxidation, indicating that PC content is not a reflection of the peroxidizability of lipids in either tissue [43]. Additionally, the n-3 fatty acids in fish oil are highly polyunsaturated and are therefore more readily oxidized, leading to higher lipid peroxidation, as observed in the Sen et al (1997) study. Furthermore, rats co-supplemented with fish oil and vitamin E exhibited significant decreases in both lipid peroxidation and protein oxidation levels in both liver and muscle [43]. Tissue levels of vitamin E in the liver were 20% higher in the rats co-supplemented with fish oil and vitamin E, as opposed to soy oil and vitamin E [43], demonstrating that with a higher intake of highly oxidizable oils, there is a concomitant higher intake of antioxidants. In accordance with Sen et al (1997), Chautan et al (1990) found a 4-fold increase in vitamin E in heart membranes of rats supplemented with PUFAs. Thus, PC content is not only dependent on the PI or UI of the oil, but also on the target tissue (brain, heart, liver and skeletal muscle) and the antioxidant content within the oil or additional antioxidant supplementation.

In our diseased animal model, the level of MDA does not reflect the level of PC under CR. It is possible that the negative energy balance as a result of CR had a greater effect on lipids than on protein in the G93A mice. In support of this, Fergani et al (2007) commented on how the defective energy metabolism in female G86R mice, possibly due to skeletal muscle hypermetabolism, leads to increased peripheral use of lipids, signifying a greater reliance on lipids in mSOD1 mouse models [44]. Similarly, short-term fasting in men and women demonstrates a greater reliance on lipids. Basal lipolytic rates during a 14-h fast were increased in women vs. men, but this sexual dimorphism was attenuated at 22-h [45]. Furthermore, female Wistar rats subjected to short-term fasting for 3 or 6 d utilize more fat and maintain lean body mass more efficiently than male rats under the same food restriction [46]. The increase in lipid usage under fasting or food restriction is not only observed in mSOD1 mice, but in humans and non-mSOD1 models as well. Furthermore, the sex difference inherent under energy restriction, with females using more fat vs males, is also conserved in humans and rats. Thus, the sex difference observed in our study, with females having greater levels of lipid peroxidation than males, may be due to the increased lipid usage in female vs. male mSOD1 mice.

We ascertained that females had significantly higher levels of MDA in the red gastrocnemius (32%, P = 0.042), but that there were no sex differences in MDA in the white gastrocnemius or in PC levels for either muscle. This finding is interesting since longevity studies in females and males have shown that females have less oxidative stress than males [47], [48]. However, female Wistar rats had higher basal levels of H_2_O_2_ production, as well as increased GPx activity in liver mitochondria, compared with their male counterparts [49]. In this study, MDA was higher in CR female red gastrocnemius, most likely due to the greater presence of mitochondria in this highly oxidative tissue [50].

In accordance with the increase in MDA levels, there was a substantial upregulation in antioxidant enzymes, MnSOD (red gastrocnemius, 4-fold; white gastrocnemius, 2.2-fold) and Cu/Zn-SOD (red gastrocnemius, 2.6-fold) in CR vs. AL females only. This protective response in females is consistent with studies showing that female rat liver mitochondria exhibit higher antioxidant gene expression (MnSOD, 2.7-fold; and GPx, 2.6-fold) [47] compared with males, and that this sexual dimorphism is conserved under CR when analyzing enzyme activity (MnSOD, 1.4-fold; and GPx, 1.3-fold) [49]. However, in spite of the compensatory increase in the protein content of these enzymes, the CR mice in our study reached clinical onset faster than AL mice. This may indicate that the protective antioxidant response was incomplete (i.e. partial), and hence insufficient to mitigate the CR-induced increase in lipid peroxidative damage. Alternatively, perhaps the increase in these enzymes is a potential reason why PC levels were decreased under CR [49], [51].

Moreover, since basal enzyme activity of MnSOD (4–5-fold), Cu/Zn-SOD (7–10-fold) and catalase (2-fold) are substantially elevated in response to increased basal levels of oxidative stress in G93A mice, any further upregulation in these enzymes may be minimal, and hence may not confer a significant benefit [32]. The antioxidant enzyme activity of MnSOD in rat liver mitochondria increased by 1.4-fold in females vs. males under CR (60% of AL) [49], while cardiac Cu/Zn-SOD activity increased by 1.3-fold in rats under CR (60% of AL) [52]. In male type 2 diabetic fatty rats, CR (30% of AL) increased total SOD activity by 2.3-fold vs. AL rats [53]. This increase is somewhat similar to what we observed in MnSOD protein content upregulation in the white gastrocnemius (2.2-fold) and in Cu/Zn-SOD protein content upregulation in the red gastrocnemius (2.6-fold). The marked increase in MnSOD in the red gastrocnemius (4-fold), although more than 2-fold higher than its reported activity in rat liver mitochondria [49], is most likely a compensatory upregulation to the 2-fold increase in MDA observed in the tissue of this diseased animal model. However, catalase enzyme activity was unchanged in our study, which is in accordance with a CR study (60% of AL) in rats that found no difference in cardiac catalase enzyme activity [52]. Although catalase activity in skeletal muscle is somewhat minimal in comparison to other tissues [41], its lack of increase may have added to the elevated lipid peroxidation in the CR mice. The increase in oxidative stress, as measured by MDA, may be the reason for the faster clinical onset and shorter lifespan observed in the CR mice.

Further support for the detriment imposed by the elevated oxidative stress in this model, is the compensatory upregulation in UCP3 protein content we observed in CR females. UCPs are mitochondrial anion-carrier proteins located in the inner mitochondrial membrane and are activated by free radicals and free fatty acids [21]. In physiological situations of oxidative stress, superoxide can induce the activation of UCP3, thus regulating mitochondrial uncoupling, which decreases the electrochemical gradient inside the intermembrane space, subsequently reducing free radical production by the ETC [21]. In turn, UCP3 uncoupling is reduced in a feedback loop. UCP3 gene expression is up-regulated before the onset of disease (90 d) in skeletal muscle of G86R mice, as well as in SALS patients [54]. UCP3 upregulation seems to be a protective response to heightened oxidative stress [54]. It is interesting that this upregulation is specific to skeletal muscle, which exhibits heightened oxidative stress in the G93A mouse [32], and also presents with a reduction in ATP levels through a decrease in the mitochondrial respiratory control ratio [54]. We have shown that UCP3 protein content is elevated in CR female skeletal muscle. This finding is in line with studies showing an association of CR with increased levels of UCP3. UCP3 protein content in rat muscle mitochondria increases in response to long-term CR (60% of AL) [55], as well long-term CR (∼60% of AL) increased UCP3 mRNA content in white adipose tissue by 2-fold [56]. These findings, in conjunction with our observed increase in UCP3 in CR females, are consistent with UCP3's role as a compensatory protective mechanism against free radical species [56]. However, there was no change in UCP3 protein content in CR males, which further supports the premise that the G93A female sex is protective under CR. The failure to upregulate UCP3 may have exacerbated disease outcomes in CR males, making males more vulnerable to the damaging effects of oxidative stress.

As previously mentioned, the protective ability of CR females to upregulate antioxidant enzymes and UCP3 was insufficient to delay the possible oxidative stress-induced decrease in life span in these animals. The role of oxidative stress in the pathogenesis of ALS is well-supported [57], [58]. However, neuroinflammation has a strong role in ALS through the pathological hallmark of microglial proliferation [59], [60], and neuroinflammation, which can be both a cause and consequence of enhanced free radical production [61]. TNF-α is an inflammatory cytokine that can activate an extrinsic, caspase-mediated apoptotic pathway upon binding to its cell-surface receptor [62]. This pathway involves the cleavage of caspase 8, which can cleave Bid (an antiapoptotic protein) and subsequently stimulate Bax to induce the release of cytochrome *c*, and cause cell death [62]. The detrimental role of TNF-α in ALS is supported by the use of thalidomide and lenalidomide to successfully inhibt TNF-α production [63], [64] and significantly increase lifespan in G93A mice [65].

Under normal conditions, protein levels of TNF-α are increased in the spinal cords of 80 d and 120 d old G93A mice, suggesting that inflammatory pathways are activated prior to onset and during the symptomatic stage of disease [66]. When life-long CR (26 mo, 60% of AL) was applied to rats, the age-associated increase in plasma TNF-α was attenuated, signifying CR's protective anti-inflammatory role [67]. However, we ascertained that the protein content of TNF-α was significantly higher (3.5-fold) in the *quadriceps* of CR vs. AL G93A mice, with no sex differences. The CR-induced elevation in this inflammatory cytokine predicts an increase in apoptotic pathways [62]. Thus, in the G93A mouse, energy restriction not only increases lipid peroxidation, but inflammation as well, potentially initiating a caspase-cascade leading to cell death.

One of the novel aspects of this study is the CR-associated increase in MDA and TNF-α. The elevated oxidative stress and inflammation present in the skeletal muscle of G93A mice suggest that this diseased mouse model lacks an adequate defense system to respond to cellular insults, such as oxidative stress and inflammation. Hsps are molecular chaperones which assist in the support of other proteins, including maintenance of proper folding and protein conformation during conditions of stress [68], [69]. Motor neurons are more vulnerable to stress than neurons in the hippocampus, cerebral cortex and substantia nigra [70]. This has implications for disease pathogenesis in ALS, as motor neurons have a reduced ability to upregulate Hsp70 in conditions of stress, making these neurons more susceptible to cellular insults [71]. Hence, when motor neurons were transfected with mSOD1, this resulted in the formation of toxic aggregates [72], [73], which were delayed by gene transfer of Hsp70 [74]. When Hsp70 is over-expressed in cerebral ischemic and ischemia-like injury models, there is a decrease in inflammation and apoptosis, as well as an increase in antiapoptotic protein Bcl-2 [75]. Similarly, exogenous delivery of Hsp70 increased lifespan in G93A mice, indicating the importance of this protein in the pathogenesis of ALS [76].

In aged rat skeletal muscle, there is a diminution of Hsp70, indicating that older animals are less protected from cellular stresses. CR (60% of AL) in old rats increased the protein content of Hsp70 by 43% vs. old AL rats, thus the effect of age on Hsp70 was attenuated by CR [77]. CR is proposed to protect neurons against age-associated stresses by inducing the activation of Hsps [78], yet the vulnerability of motor neurons in ALS disease pathology may be incapable of this protective response [71]. Furthermore, the presence of Hsps in the cytosol and intermembrane space of the mitochondria help prevent cell death. However, the binding of mSOD1 to Hsp70 may impede Hsp70's normal antiapoptotic function, potentially promoting apoptosis and making the mitochondria more susceptible to stress [79].

In our study, both female and male CR mice had significantly decreased levels of Hsp70 in the *quadriceps* compared to AL mice. CR male mice had significantly less Hsp70 protein content than CR females, indicating that they are less protected from cell stress. It is possible that the failure of G93A mice to upregulate Hsp70 led to the faster clinical onset and disease progression observed under energy restriction and that once again, males were poorly defended against the heightened lipid peroxidation and inflammation compared to females. Our results coincide with the previous studies mentioned, which illustrate the reduced ability of motor neurons to upregulate Hsp70 under stress conditions [71], and the inhibition of Hsp70's antiapoptotic role through its binding with mSOD1 [79].

The significant decrease in Hsp70, together with the heightened peroxidative damage and inflammation we observed in G93A mice, may be the catalysts for apoptotic cell death under CR. In symptomatic G93A mice, Bax levels are increased and Bcl-2 levels are decreased in the spinal cord as compared to age-matched nontransgenic controls [80]. Bcl-2, an antiapoptotic protein, and Bax, a proapoptotic protein, can moderate levels of apoptosis by preventing or promoting the release of cytochome c [62]. The elevation in TNF-α protein content observed in CR mice may elicit cell death through the activation of apoptotic proteins [62]. Furthermore, the decrease in Hsp70 in the CR mice is indicative of increased apoptosis, since the presence of Hsp prevents the recruitment of caspase 9 to the apoptosome [81].

In our study, there was a significant increase in Bax protein content in CR vs. AL mice. Normally, CR functions to decrease oxidative stress by inhibiting apoptosis [62] through a reduction in Bax (in the aging mouse heart) [82] and elevation in Bcl-2 (in the aging rat kidney) [83]. We observed that the ratio of Bax/Bcl-2 also increased, signifying enhanced cell death in the skeletal muscle of CR mice by increasing the activation of mitochondrial permeability transition pores [84]. Moreover, there was no change in Bcl-2 protein content between CR and AL mice. Our finding corresponds with another study which did not find an effect of CR (70% of AL) on Bcl-2 gene expression in male rat hepatocytes [85]. The unaltered protein content of Bcl-2 suggests a lack of protection from apoptosis, since the over-expression of Bcl-2 prolongs lifespan in G93A mice [86]. Additionally, 2B4 cells treated with an apoptotic signal, dexamethasone, resulted in lipid peroxidative damage, which was prevented by the over-expression of Bcl-2 [87]. The elevated lipid peroxidation in our mice may be the result of an imbalance between proapoptotic and antiapoptotic proteins. This observation is consistent with the increased MDA, in conjunction with significantly elevated levels of Bax and the ratio of Bax/Bcl-2 protein content in CR vs. AL females. It is likely that the heightened lipid peroxidation led to higher levels of Bax in females, although these levels were not significantly different between CR females and males. However, the protein content of Bax/Bcl-2 in CR females is indicative of higher apoptosis. Activation of this proapoptotic protein may potentially trigger apoptotic cell death and is a possible explanation of why CR hastened the clinical onset of disease and disease progression in G93A mice

Conversely, there were no significant differences in the protein content of caspase 9, cleaved caspase 9 or the ratio of cleaved caspase 9/caspase 9. During apoptosis, translocation of Bax from the cytosol to the mitochondria in the spinal cords of G93A mice [88] triggers the release of cytochrome *c* from the mitochondria [89]. Once in the cytosol, cytochrome *c* interacts with Apaf-1 and dATP forming an apoptosome that recruits caspase 9 [90]. This initiator caspase can then activate downstream effector caspases 3 and 7 [91], which can cleave caspase 9 into its smaller 37 kDa fragment, signaling mitochondrial-dependent apoptotic cell death [88]. Guegan et al (2001) illustrated that the protein content of caspase 9 begins to decrease in the spinal cords from symptomatic G93A mice (3 mo of age) compared to nontransgenic control mice and that caspase 9 continues to decrease further in end stage G93A mice (5 mo of age). Alternatively, cleaved caspase 9 protein content begins to increase in symptomatic G93A mice, with highly expressed levels in end stage mice [88]. The increase in cleaved caspase 9 protein content signifies an elevation in apoptosis in the later stages of the disease. In our study, G93A mice were sacrificed at 99 d (∼3 mo), and while we detected both the cleaved and uncleaved forms of caspase 9, we did not observe any significant changes in these proteins. Although we have demonstrated increases in Bax protein content, the failure to obtain an increase in the ratio of cleaved caspase 9/caspase 9 between the CR and AL groups may be due to the timing of tissue collection. This is in accordance with a study which found changes in Bax protein content, but no change in caspase 3 activity with training in 3-month old rat skeletal muscle [92]. Additionally, life-long CR (60% of AL) in 26-month old rats (starting at 3.5 mo of age) did not alter caspase 9 activity in the brain compared to age-matched rats on an AL diet, despite an attenuation in DNA fragmentation with CR [93]. A similar study examining life-long CR (60% of AL) in rat showed decreased levels of cleaved caspase 3 with CR in 26-month old gastrocnemius, but no significiant differences in the protein content of caspase 9 or cleaved caspase 9 [94]. Furthermore, short-term CR (2 mo; 60% of AL) increased apoptosis in the liver of male rats in the absence of significant changes in caspase 9 or cleaved caspase 9 protein content [95]. These studies provide support that apoptosis could occur in the absence of any significant alterations in caspase 9 activity or cleaved caspase 9 protein content with CR. Thus, we show that CR increases Bax protein content, with no difference in caspase 9 or cleaved caspase 9 levels in our diseased animal model, illustrating that not every component of the mitochondrial-mediated apoptotic pathway is necessarily affected by CR.

We conclude that CR, equivalent to 60% of AL, hastens clinical onset by 2-fold, disease progression by 13-18% and endpoint by 3.1-fold. We ascertained that CR increases mitochondrial biogenesis, lipid peroxidation and inflammation, while decreasing mitochondrial oxidative capacity, cellular stress response in the G93A mouse, leading to heightened apoptosis. We postulate that these series of events may explain the faster clinical onset and shorter lifespan observed in this animal model. The substantial upregulation of antioxidant enzymes and UCP3, a marker of mitochondrial energetics, may be a protective response to the heightened oxidative damage within the CR females. In contrast, dysregulation in cellular redox status and less protection from heat shock proteins exacerbates disease outcomes in CR males. Our study is unique from other intervention studies in that we have investigated the effect of CR in vivo by analyzing anthropometric, functional, biochemical and molecular outcome measures. Ultimately, this is the first study to illustrate that CR is detrimental in G93A mice and that if we were to extrapolate our findings to patients with the disease, CR would be contraindicated.
